# Placenta percreta invading left broad ligament in a woman with twin pregnancy: A case report

**DOI:** 10.1016/j.amsu.2022.104875

**Published:** 2022-11-15

**Authors:** Shahzaib Maqbool, Iqra Zulqarnain, Imran Khan, Muhammad Farhan, Zara Noor, Mohammad Ebad Ur Rehman, Aimen Bibi, Jawad Basit, Sajeel Saeed

**Affiliations:** a Department of Gynecology and Obstetrics, Holy Family Hospital, Rawalpindi, Pakistan; b Rawalpindi Medical University, Rawalpindi, Pakistan; c Sandeman Provincial Headquarter Hospital, Quetta, Pakistan; d Quaid-e-Azam Medical College, Bahawalpur, Pakistan

**Keywords:** Placenta percreta, Broad ligament, Twin pregnancy

## Abstract

**Introduction:**

*and importance*: Placenta percreta is an abnormal of placentation disorder that causes firm and deep attachment of placenta into myometrium due to absent decidua basalis and leads to significant morbidity and mortality due to severe hemorrhage.

**Presentation of case:**

A 28-year, old women gravida 2 para 1 + 0 with previous one Lower segment caesarean section (LSCS), presented to emergency department of HFH with complaint of per vaginal bleeding. It was a twin pregnancy and was a rare case of complex placenta percreta with invasion into left broad ligament and urinary bladder in a woman having twin pregnancy. Placental invasion into bladder was diagnosed pre-operatively on USG scan, however; the broad ligament involvement was diagnosed intraoperatively. Patient underwent hysterectomy and internal iliac artery ligation to control hemorrhage soon after delivery of twins with T2 being IUD and patient shifted to ventilatory support but unfortunately due to massive hemorrhage and hemodynamic instability patient did not survive.

**Discussion:**

Placenta percreta is a subtype of placenta accreta spectrum disorder that is associated with significant morbidity and mortality depending upon nature and extent of placental invasion. Preoperative diagnosis and management can be of significant value in preventing obstetrics related morbidity. A multidisciplinary approach is required in management of such cases and due to involvement of surrounding structures including urinary bladder.

**Conclusion:**

Placenta percreta is a rare disorder of placentation that poses significant life-threatening risk of bleeding and maternal mortality and multidisciplinary approach can be of benefit in such cases.

## Highlights

• Placenta percreta is a rare disorder that poses significant life-threatening risk to maternal as well as fetal wellbeing.
• Early diagnosis can reduce mortality and morbidity.
• A multidisciplinary approach is required in management of such cases of complex nature.

## 1 Introduction

Placenta accreta spectrum is an abnormal form of placentation leading to firm attachment to the myometrium due to absence of decidua basalis [1]. Placenta accreta spectrum disorder also known as abnormally invasive placenta (AIP) consisted of three subtypes such as placenta accreta, increta, and percreta depending upon the depth of placental invasion [2]. The superficial invasion of myometrium by chorionic villi is termed as placenta accreta, extensive invasion of myometrium with chorionic villi is called as placenta increta, however, invasion throughout the layers of uterus up-to the serosa with or without involvement of contiguous structures and organs is called percreta [3].

The rate of caesarean section is on the high throughout the word from last few decades ranging from the 25–30% as compared to 5–8% [3]. Increased rate of cesarean section is the leading cause of abnormal placental invasions in next pregnancies. Percreta is the most severe form of placenta accreta with incidence of 5–7% throughout the world [4]. Placenta percreta is life threatening condition as it may leads to massive bleeding, emergency hysterectomy and significant risk of morbidity and mortality. The clinical outcome become worse, when placenta percreta coexist with placenta previa because in this condition the diagnosis is made peroperatively [5].

Placenta percreta can involve the surrounding structures most commonly, bladder and rectum. Involvement of broad ligament is very rare in placenta percreta [6]. Herein we reported a case of placenta percreta invading into the broad ligament along with bladder in a woman with twin pregnancy and previous 1 LSCS. This work has been reported in line with the SCARE 2020 criteria [7].

## 2 Presentation of case

A 28-year, old women gravida 2 para 1 + 0 with previous one Lower segment caesarean section (LSCS),3.5 years back, presented to emergency department of gynecology and obstetrics Unit of Holy Family Hospital Rawalpindi with complaint of per vaginally bleeding. Patient initially presented in District head quarter (DHQ) hospital Bagh from where she was referred to Holy Family Hospital because of twin pregnancy and preterm premature rupture of membranes (PPROM) at 29 + 2 weeks of gestation. Her last menstrual period (LMP) was on 8^th^ June 2021 making her expected date of delivery (EDD) 15^th^ March 2022. The dating scan was already performed at 13 weeks of pregnancy in the DHQ hospital Bagh which was showing diamniotic dichorionic twin pregnancy with crown rump length (CRL) of twin l was 63.7mm and CRL of twin 2 was 65.4mm with positive cardiac activity of both twins. Two placental masses were noted at the time of dating scan confirming chorionicity and amnionicity.

**Fig. 1:**
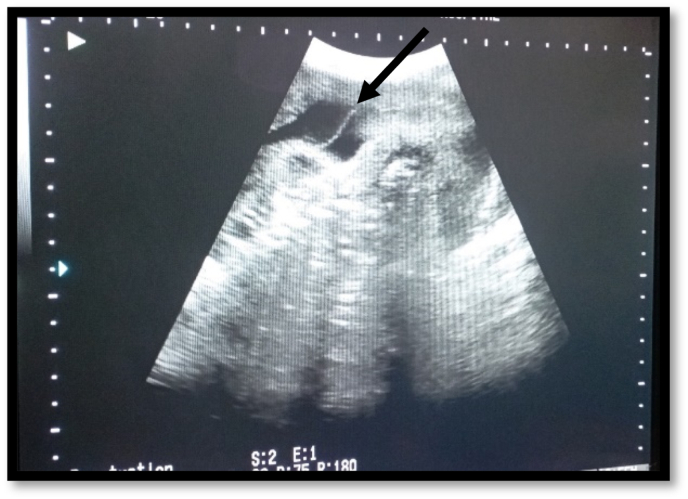
Showing the dividing members of dichorionic diamniotic twin pregnancy (arrow head).

At 29 + 2 weeks patient presented to emergency department of gynecology and obstetrics unit of Holy Family Hospital, Rawalpindi with complaint of painless mild per-vaginal bleeding along with preterm premature rupture of membranes. Patient was vitally stable with the following vitals; blood pressure (B.P) of 110/70, pulse rate (PR) of 84 and saturation of 99% at room air and patient was afebrile. On general physical examination patient was not pallor, no cyanosis, clubbing, jaundice were observed. On per abdominal examination the abdomen was soft non-tender and symphysis-fundal height corresponds to 32cm, fetal lie could not assess due to multiple gestation. The 29 + 2 weeks scan performed in the DHQ was not only showing the twin pregnancy, but also showing the suspicion of placenta Previa grade II, therefore per vaginally the examination was not performed at the time of presentation to avoid any life-threatening bleeding. Patient scan was also performed in emergency department what was showing twin alive gestation, the presentation and lie of T1 and T2 was longitudinal cephalic. The fetal movements and cardiac activity of both twins (T1&T2) was positive and liquor volume was also adequate. 1st placenta was anterior, low-lying grade III (major degree Previa), while 2nd placenta was posterior as the twin pregnancy was dichorionic diamniotic the dividing member was also seen on USG scan as shown in Fig. 1. Similarly, biometric profile of both twins is shown in Table 1.

**Table 1:** Showing Biometric profile of twin 1 (T1) and twin 2 (T2).

Parameters of twin 1	Values	Corresponding gestational age
**BPD**	72mm	28 + 2 weeks
**FL**	55mm	29 + 0 weeks
**Parameters Of twin 2**	**Values**	**Corresponding gestational age**
**BPD**	76mm	30 weeks
**FL**	55mm	29 + 0 weeks

BPD: Biparietal diameter, FL: Femur length.

**Fig. 2:**
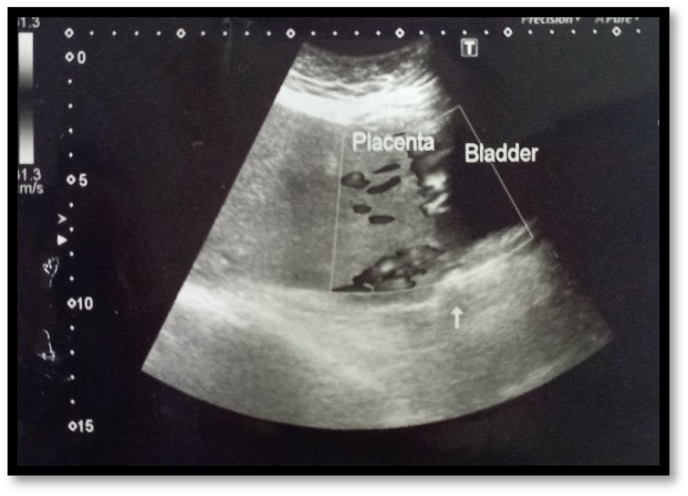
Showing the placental invasion into urinary bladder.

Patient was then admitted to antenatal ward of obstetrics and gynecology unit I of Holy Family Hospital, Rawalpindi where Patient's initial workup was started and baseline investigations were performed. On laboratory investigations, her hemoglobin level (Hb) was 11.2g/dl, total leukocyte count was 9.8x10^9^ and platelet count was 242 × 10^9^/L was observed. Patient's liver function tests, Renal function tests and coagulation profile was also with in reference range. Patient's anemia workup was also done which showed serum ferritin levels of 7.1ng/ml (females: 10.0–73.3) and Hb electrophoresis was also performed which was also normal (Hb A1: 96.9%, Hb F: 0.7%, Hb A2: 2.4%). Antenatal care progressed normally without any significant complaint and patient remain vitally stable. She had a repeat scan at 32 + 3 weeks which was showing longitudinal cephalic presentation of T1& T2 was breech, however, the gross body movements and fetal cardiac activity of both twins was positive and the parameters of T1 were corresponding to 31 + 4 weeks and T2 parameters were corresponding to 30 + 1 weeks with adequate liquor and no gross anomaly on anomaly scan. The doppler USG was also performed which showed placental invasion into bladder as shown in Fig. 2. The fetal monitoring through cardiotocography (CTG) was started twice daily to look for fetomaternal surveillance.

**Fig. 3:**
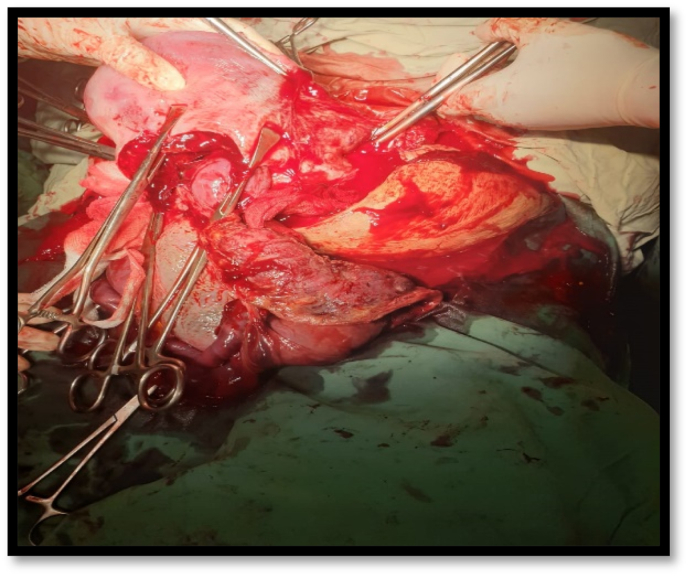
Showing the placenta percreta invading the left broad ligament and bladder in complex manner.

At 34 + 2 weeks, patient complained of decrease fetal movements, biophysical profile performed which was 6/10 at the moment and cardiotocograph showed category III changes. Patient was immediately prepared and proceeded for emergency lower segment cesarean section. Senior anesthetist, senior obstetrician, urologist was involved due to placenta percreta. Just before undergoing emergency LSCS, fetal cardiac activity of T2 was confirmed on ultrasound which was negative. Patient and attendants were counseled regarding nature of severity of condition and consent of hysterectomy and ICU/ventilator support was taken. Patient was given general anesthesia scrubbed and draped. A midline incision was given and the twins were delivered. T1 was delivered with good APGAR score 2.2kg weight, pediatrics assessment done and shifted to NICU while T2 was IUD. Per-operatively a complex placental invasion into bladder and left broad ligament was observed validating the presence of placenta percreta as shown in Fig. 3 peripartum hysterectomy was performed to prevent blood loss, bladder was also repair. The removed specimens (uterus, cervix and placenta) were sent for histopathology. The patient was than transfused with 2 units of packed red cells and 4 units of fresh frozen plasma (FFP) and patient was closed in reverse order and drain was placed inside for any collection and finally patient was shifted to surgical intensive care unit (SICU). At the time of shifting to SICU patient was vitally stable.

In the same evening patient become vitally unstable with reduced Hb 8.0 mg/dl and 500ml drain output. The patient was again shifted to operation theater and reopen laparotomy was done which was showing 1.5 L hemoperitoneum and at the same moment internal iliac artery was ligated and small bleeders from omentum were also secured and after patient was again transfused with 2 RCC and after becoming vitally stable the patient was again shifted to SICU and strict vital monitoring was ensured and a good antibiotic cover was also given.

## 3 Discussion

The combination of placenta percreta is not a common occurrence in obstetrics, and it is one of the deadly complications encountered by obstetrician during obstetrical surgeries. It is frequently associated with massive life-threatening hemorrhage and its related complications, multiple viscera injuries due to frequent involvement of bladder and bowel [6]. The incidence of placenta percreta is increasing over the years. It was first reported by Irving and Hertig in year 1937 [8], and in 1977 a very low incidence of 1 in 7000 cases was reported by Breen et al. [9]. But a study conducted by Wu et al. have reported an alarming rise in cases reporting incidence of 1 in 533 cases as reported in year 2005 [4]. The paramount increase in the cases of placenta percreta is attributed to the rising trend of c-sections. The higher the number of previous c-sections, the higher the risk of placenta percreta development [2].

The risk factors associated with placenta percreta are previous ceserean section, multiple pregnancies, advanced maternal age, placenta previa, dilatation and curettage, endometritis, and repetitive abortions [5]. This case was also true depiction of multiple pregnancies associated placenta percreta as this was a twin pregnancy. The diagnostic value of sonography in prenatal diagnosis of placenta percreta is uncertain with positive predictive value of 78% and negative predictive value of 94% as been reported by Finberg et al. [10]. If USG findings are inconclusive than magnetic resonance imaging (MRI) can be of the valuable adjunct to USG in prenatal diagnosis of placenta percreta [10]. But, in this reported case the urinary bladder invasion was even reported on the Doppler scan as shown in Fig. 3. The placental invasion into broad ligament is a rare finding as occur in our case but pre-operatively the USG findings were only just suggestive of bladder invasion with no evidence of broad ligament invasion as observed intraoperatively.

Various treatment options have been suggested for placenta percreta but, surgical intervention is considered as the first line treatment option because in 93% of the cases hysterectomy need to done to control bleeding as it happened in this reported case [11]. However, the conservative management with use of methotrexate an antineoplastic drug, is also another option but studies have also contradicted the use of methotrexate as it would ultimately results in hysterectomy due to postpartum bleeding [12]. In the same vein, the hysterectomy is the only life saving intervention to control internal bleeding. Bilateral internal iliac ligation is also the option as we did in our case because the placental invasion into the broad ligament stretches the peritoneal part of the ligament with ultimate outcome of massive intraperitoneal bleeding [13], leading to death and these findings were in concordance with the case we represented. The management of complex natured placenta percreta required multidisciplinary approach that involved obstetrician, gynecologist, anesthesiologist, neonatologist, urologist, radiologist and involvement of blood bank physician [14]. Studies have reported the favorable outcomes resulted from involvement of multidisciplinary team approach [15,16]. This particular case was also managed by multidisciplinary team approach, however; this was a case of twin pregnancy that poses a bit more difficulty in management. This particular topic of placenta percreta still in need to be studied in more depth and also required more insight of world obstetricians for innovative management approach.

## 4 Conclusion

Herein a rare case of placenta percreta has been reported in patient with previous I LSCS. Placenta percreta with invasion of urinary bladder and parametrium is a life-threatening condition that possess significant maternal morbidity and mortality risk. This case highlights a rare happening of placenta percreta invasion in to bladder and left broad ligament in a woman with twin pregnancy. The placenta removal was associated with significant obstetric hemorrhage and bladder injury leading to hysterectomy and bilateral internal iliac artery ligation. But unfortunately leading to maternal mortality due to significant blood loss. This would highlight the severity of disease condition and will help to look for better prenatal screening, diagnosis and to get insight of better site and plan for delivery.

## Ethical approval

Not applicable.

## Sources of funding

None.

## Author contribution

Shahzaib Maqbool and Iqra Zulqarnain were involved in patient care during hospitalization and surgery and in drafting the article. Imran Khan, Mohammad Ebad Ur Rehman, Aimen Bibi, Jawad Basit and Sajeel Saeed were involved in drafting and revising the article to the final form as submitted. Zara Noor and Muhammad Farhan were involved in patient care during hospitalization and in revising the article to the final form as submitted.

## Registration of research studies

1. Name of the registry: Not applicable.

2. Unique Identifying number or registration ID: Not applicable.

3. Hyperlink to your specific registration (must be publicly accessible and will be checked): Not applicable.

## Guarantor

Muhammad Farhan.

Mohammad Ebad Ur Rehman.

## Provenance and peer review

Not commissioned, externally peer-reviewed.

## Declaration of competing interest

The authors have no conflicts of interest.

## References

[1] Wortman A.C., Alexander J.M. (2013 Mar). Placenta accreta, increta, and percreta. Obstet. Gynecol. Clin. N. Am..

[2] Collins S.L., Ashcroft A., Braun T., Calda P., Langhoff-Roos J., Morel O., Stefanovic V., Tutschek B., Chantraine F. (2016 Mar). European Working Group on Abnormally Invasive Placenta (EW-AIP). Proposal for standardized ultrasound descriptors of abnormally invasive placenta (AIP). Ultrasound Obstet. Gynecol..

[3] Honig A., Rieger L., Thanner F., Eck M., Sutterlin M., Dietl J. (2005 Oct). Placenta percreta with subsequent uterine rupture at 15 weeks of gestation after two previous cesarean sections. J. Obstet. Gynaecol. Res..

[4] Wu S., Kocherginsky M., Ju Hibbard (2005 May). Abnormal placentation: twenty-year analysis. Am. J. Obstet. Gynecol..

[5] Abinader R.R., Macdisi N., El Moudden I., Abuhamad A. (2021 Nov 27). First-trimester ultrasound diagnostic features of placenta accreta spectrum in low-implantation pregnancies. Ultrasound Obstet. Gynecol..

[6] Lin C.C., Adamczyk C.J., Montag A.G., Zelop C.M., Snow J.C. (1998 Sep). Placenta previa percreta involving the left broad ligament and cervix. A case report. J. Reprod. Med..

[7] Agha Riaz A. (2020). The SCARE 2020 guideline: updating consensus surgical CAse REport (SCARE) guidelines. Int. J. Surg..

[8] Irving F.C. (1937). A study of placenta accreta. Surg. Gynecol. Obstet..

[9] Breen J.L., Neubecker R.O., Gregori C.A., Franklin J.E. (1977 Jan 1). Placenta accreta, increta, and percreta. A survey of 40 cases. Obstet. Gynecol..

[10] Finberg H.J., Williams J.W. (1992 Jul). Placenta accreta: prospective sonographic diagnosis in patients with placenta previa and prior cesarean section. J. Ultrasound Med..

[11] Kingdom J.C., Hobson S.R., Murji A., Allen L., Windrim R.C., Lockhart E., Collins S.L., Majd H.S., Alazzam M., Naaisa F., Shamshirsaz A.A. (2020 Sep 1). Minimizing surgical blood loss at cesarean hysterectomy for placenta previa with evidence of placenta increta or placenta percreta: the state of play in 2020. Am. J. Obstet. Gynecol..

[12] Matsuzaki S., Yoshino K., Endo M., Kakigano A., Takiuchi T., Kimura T. (2018 Mar). Conservative management of placenta percreta. Int. J. Gynecol. Obstet..

[13] Hussein A.M., Dakhly D.M., Raslan A.N., Kamel A., Abdel Hafeez A., Moussa M., Hosny A.S., Momtaz M. (2019 Oct 18). The role of prophylactic internal iliac artery ligation in abnormally invasive placenta undergoing caesarean hysterectomy: a randomized control trial. J. Matern. Fetal Neonatal Med..

[14] Nieto Albaro José (2020). Placenta accreta: importance of a multidisciplinary approach in the Colombian hospital setting. J. Matern. Fetal Neonatal Med.: Off. J. Eur. Ass. Perinatal Med..

[15] DeSimone Robert A. (2018). Transfusion medicine in a multidisciplinary approach to morbidly adherent placenta: preparing for and preventing the worst. Transfus. Med. Rev..

[16] Lee Paula S. (2017). Multidisciplinary approach to manage antenatally suspected placenta percreta: updated algorithm and patient outcomes. Gynecol. Oncol. Res. Pract..

